# Low thalamic activity during a digit-symbol substitution task is associated with symptoms of subjective cognitive decline

**DOI:** 10.3389/fpsyt.2023.1242822

**Published:** 2023-09-06

**Authors:** Akiko Mizuno, Helmet Talib Karim, Maria J. Ly, Brian J. Lopresti, Ann D. Cohen, Areej A. Ali, Chester A. Mathis, William E. Klunk, Howard J. Aizenstein, Beth E. Snitz

**Affiliations:** ^1^Department of Psychiatry, University of Pittsburgh, Pittsburgh, PA, United States; ^2^Department of Bioengineering, University of Pittsburgh, Pittsburgh, PA, United States; ^3^Department of Neuroscience, University of Pittsburgh, Pittsburgh, PA, United States; ^4^Department of Radiology, University of Pittsburgh, Pittsburgh, PA, United States; ^5^Department of Neurology, University of Pittsburgh, Pittsburgh, PA, United States

**Keywords:** subjective cognitive decline, fMRI, digit-symbol substitution task, executive function, amyloid

## Abstract

**Introduction:**

Subjective cognitive decline (SCD) may represent the earliest preclinical stage of Alzheimer's Disease (AD) for some older adults. However, the underlying neurobiology of SCD is not completely understood. Since executive function may be affected earlier than memory function in the progression of AD, we aimed to characterize SCD symptoms in terms of fMRI brain activity during the computerized digit-symbol substitution task (DSST), an executive function task. We also explored associations of DSST task performance with brain activation, SCD severity, and amyloid-ß (Aß) load.

**Methods:**

We analyzed data from 63 cognitively normal older individuals (mean age 73.6 ± 7.2) with varying degree of SCD symptoms. Participants completed a computerized version of DSST in the MR scanner and a Pittsburgh Compound-B (PiB)-PET scan to measure global cerebral Aß load.

**Results:**

A voxel-wise analysis revealed that greater SCD severity was associated with lower dorsomedial thalamus activation. While task performance was not associated with brain activation nor Aß load, slower reaction time was associated with greater SCD severity.

**Discussion:**

The observed lower dorsomedial thalamus activation may reflect declining familiarity-based working memory and the trans-thalamic executive function pathway in SCD. SCD symptoms may reflect altered neural function and subtle decline of executive function, while Aß load may have an indirect impact on neural function and performance. Self-perceived cognitive decline may serve as a psychological/subjective marker reflecting subtle brain changes.

## Introduction

Subjective cognitive decline (SCD) is common among older adults and a possible clinical state proposed for research that may represent the earliest detectable sign of cognitive decline in aging in some individuals and may predict the future development of Alzheimer's disease (AD) (1). Individuals with SCD have elevated self-perceived cognitive decline or concerns regarding their cognitive function but perform within the normal range on standard neuropsychological tests. For some individuals with SCD, their symptoms predict a higher risk for future mild cognitive impairment (MCI) and AD diagnosis (2, 3). The primary symptoms of SCD are self-perceived decline or complaints regarding memory. While objective memory impairment represents the classical hallmark of AD, multiple studies have suggested that executive function may decline (4, 5), possibly even prior to memory impairment (6). Investigating neural correlates of executive functioning among individuals with SCD may thus improve our understanding of potential early signs and symptoms of cognitive decline and their underlying neural mechanisms.

Executive function encompasses higher-order cognitive processes that allow for complex mental operations, such as attention, planning, monitoring, inhibition, task switching, and working memory (7). SCD has been reported to be associated with lower executive function performance, resembling deficits seen in AD (8–10). Previous neuroimaging studies with executive function tasks in SCD have yielded somewhat mixed results of hyper- and hypoactivation (10–12). In contrast, AD-related functional and structural pathological changes in executive function and associated brain networks are well-characterized. For example, impaired executive function is associated with reduced metabolism in the cognitive control network (i.e., dorsolateral prefrontal, anterior cingulate, and parietal cortex) (13–16) and decreased structural integrity of this network (17, 18). Individuals with MCI had greater activation during executive function tasks in the cognitive control network in comparison with those without cognitive impairment (19, 20). This heightened activation is often interpreted as a compensatory process to maintain cognitive performance in response to pathological changes in the brain such as cerebral amyloid-beta (Aβ) load, atrophy, and white matter structural damage. Given that SCD may precede MCI (1), mirroring the relationship between MCI and AD, it is possible that the hyperactivation in the cognitive control network observed in SCD may underlie the suboptimal yet maintained performance of executive functioning in an AD pathway, at least for some. However, the literature on neuroimaging studies involving SCD remains limited and inconclusive, displaying both hyper- and hypoactivation patterns. These inconsistencies could be attributed to the inherent complexity of “executive functions,” which comprise various intricate subdomains. To address this gap, we aimed to enhance our understanding of the relationship between executive functioning and SCD symptoms using a different task—the digit-symbol substitution test (DSST). This task may provide additional insights into the underlying mechanisms of executive dysfunction in SCD and its potential ties to AD-related pathways.

In the present study, we employed a computerized version of the DSST [adapted from the Weschler Adult Intelligence Scale-Revised (WAIS-R)] (21). The WAIS-R DSST, a paper-and-pencil test, is a commonly used neuropsychological measure sensitive to age-related cognitive decline (22) and pathology (23) and also shows associations with the functional ability to complete everyday tasks (24). The DSST is “polyfactorial” (24), requiring multiple cognitive operations for successful performance (25), including working memory, cognitive control, attention, and processing speed. Previous fMRI studies have used computerized versions adapted for the fMRI scanner procedures, as in the present study, and they have shown DSST-induced activations in the frontoparietal cognitive control network (26–29), supporting the notion that DSST involves multiple cognitive processes. The fMRI version of the DSST of the present study (28, 29) was designed to induce activations related to working memory and attentional control (see task description below).

Another understudied area in SCD research is the relationship among SCD symptoms, neural function (e.g., hypo- or hyperactivation), and Aß load. A previous study from our group assessed the relationship between cerebral Aß load and DSST-induced activation among a partially overlapping sample (57% of overlap) of cognitively normal older adults (29). This study found that greater Aß deposition was associated with greater activation in the insula, inferior frontal gyrus, precuneus, calcarine, and middle temporal regions, indicating a possible compensatory hyperactivation corresponding to Aß load. Another study from our group in the same sample as the current study (30) found the previously reported positive association between Aß load and SCD symptom severity (31, 32). The relationship between SCD symptom severity and DSST-induced activation with this task has not been tested. Building on these results, we hypothesized that greater SCD symptom severity would be associated with greater cortical DSST-induced activations, since SCD symptoms correlate with Aß, which is correlated with DSST activation. In addition to the main hypothesis, we explored the relationship between DSST-induced activation and task performance (i.e., reaction time of the in-scanner DSST task). We hypothesized that both greater SCD symptom severity and Aß load (i.e., known AD risk factors) would be associated with lower DSST task performance (i.e., slower reaction time).

## Methods

### Participants

Data from 66 cognitively normal older adults (average age = 73.6 ± 7.2) with a varied range of SCD symptom severity were analyzed from two study settings: the University of Pittsburgh Alzheimer's Disease Research Center (ADRC) study on SCD (*n* = 21) and a volunteer-based neuroimaging community study of aging (*n* = 45). The diagnostic criteria of SCD were broadly aligned with the broad symptomatic definition of pre-MCI SCD by the Subjective Cognitive Decline Initiative (SCD-I) working group (1), including (1) self-perceived cognitive decline, (2) normal neuropsychological testing (described in the next section), (3) no MCI or dementia diagnosis, and (4) no major psychiatric/neurologic disease. Additional inclusion criteria for the ADRC SCD study were age 50 and older, self-referred (i.e., seeking clinical evaluation) because of subjective cognitive concerns, normal on neuropsychological testing (described in the next section), and English fluency. Inclusion criteria for volunteers from the surrounding regions of the University of Pittsburgh were age 65 and older, normal objective cognitive function, and English fluency. The following exclusion criteria applied to both groups: MCI or dementia diagnosis, history of major neurologic disease (e.g., Parkinson's disease, Huntington's disease, multiple sclerosis), lifetime history of schizophrenia, manic-depressive disorder, or schizoaffective disorder, current substance abuse or dependence, current medical conditions/medications that may affect cognitive function (e.g., recent brain surgery, chronic renal failure, severe pulmonary disease), and contraindications for MRI or PET scans. Additionally, in the community sample, we excluded individuals with significant psychoactive medication use (e.g., narcotics, benzodiazepines, and sedatives) or current clinical depression [defined as a score of 15 on the Geriatric Depression Scale (GDS)]; however, we did not exclude individuals with a history or current anxiety disorders. Each participant underwent a comprehensive multi-domain neuropsychological assessment by trained clinical staff, and the assessments were reviewed in a diagnostic consensus conference that involved at least two ADRC investigators (authors BS and WK) and required at least two-person agreement. The ADRC diagnostic consensus conference is larger and multi-disciplinary. The University of Pittsburgh Institutional Review Board reviewed all protocols in this study, and all participants provided written consent prior to participation.

One memory clinic patient and one community volunteer were excluded due to poor task performance (described below), and one community volunteer was excluded due to excessive head motion in the MR scanner (more than 20% of resting state data identified as head jerks), yielding a final sample of *n* = 63 [83% overlap of cognitively normal older adults (57% of total) with our previous report] (29). Since we combined the participants from two recruitment sources, we controlled for this group variable in our analyses.

### Neuropsychological battery and SCD measures

The same neuropsychological battery was utilized in both samples. Normal cognitive function was defined as having no more than two scores that were below one standard deviation age- and education-adjusted norms on the following neuropsychological battery: global cognitive function [Mini-Mental State Examination (33)]; *memory* [Word List Learning from the Consortium to Establish a Registry in Alzheimer's Disease battery (34), modified Rey-Osterrieth immediate and delayed recall (35)] *language* [Boston Naming test (36), and Letter/Category Fluency (37)]; *visuospatial abilities* [Modified Block Design, Digit Spans Forward/Backward (21)], and *executive function*s [Trail Making Test (Trails B-A) (37), a paper-and-pencil version digit symbol (21)].

As detailed previously in Snitz et al. (38), these scores were reviewed at a diagnostic consensus conference as part of the ADRC protocol. The NEO Five-Factor Inventory 3 (39) was administered to assess neuroticism, which has often been associated with SCD (40). Depressive symptoms were measured by the Geriatric Depression Scale (GDS) (41).

The Memory Functioning Questionnaire (MFQ) (42), the Cognitive Failures Questionnaire (CFQ) (43), and the Subjective Cognitive Complaint Scale (SCCS) (44) were utilized to assess the severity of SCD symptoms. The MFQ is a 64-item scale assessing six factors: retrospective functioning, e.g., “How is your memory compared to … 1, 5, 10 years ago?”; frequency of forgetting, e.g., of names, faces, appointments, and where you put things; forgetting during reading; remembering past events; and seriousness (i.e., how “serious” a problem is forgetting names, faces, and appointments). The CFQ is a 25-item scale assessing the likelihood of committing errors during everyday tasks, e.g., failing to notice signs on the road or remembering what was intended to buy at the store. The SCCS is a 24-item scale assessing common memory and other cognitive complaints across the spectrum from normal aging to MCI and mild dementia, e.g., worsening of remembering things that happened a few days ago, how to use appliances, and understanding what people say, assessing a more extended range of subjective symptom severity than the other two scales.

The standardized scores of each of the MFQ, CFQ, and SCCS measures were first calculated using the adjusted means from previously published studies (38, 45, 46) and standard deviations from the current participants' responses, indicating an age-appropriate average score of zero. The MFQ score was then inverted to indicate that higher scores on all three measures represented worse SCD symptom severity. The mean of three *Z*-scores comprised a composite SCD symptom score, which was included in analyses to indicate “SCD symptom severity.” We confirmed the high internal consistency among the three SCD measures. Composite reliability computed by confirmatory factor analysis [“lavaan” R package (https://cran.r-project.org/web/packages/lavaan/index.html)] was 0.89 (standardized factor loadings β = 0.85, 0.92, 0.78, respectively, Supplementary Figure S1) with a suggested threshold >0.70 (47).

### PET data acquisition

As detailed in Wilson et al. (48), [^11^C]PiB was synthesized by a simplified radiosynthetic method based on the captive solvent method. Fifteen 15 mCi of high-specific activity [^11^C]PiB (~2.1 Ci/μmol at end-of-synthesis) was injected intravenously over 20 s prior to scan acquisition. In order to correct for attenuation, a 10–15 min windowed transmission scan was acquired, followed by a 20-min emission scan (4 × 300 s frames) beginning 50-min post-injection.

These scans were acquired on a Siemens/CTI ECAT HR+ scanner (Siemens Medical Solutions, Knoxville, TN) in 3D mode [63 axial imaging planes, field-of-view (FOV) 15.2 cm, in-plane resolution 4.1 mm full-width at half-maximum, at FOV center, axial slice width 2.4 mm], which is equipped with a neuro-insert to reduce scattered photon contribution. PET emission data were reconstructed using filtered back projection and included standard corrections for attenuation, scatter, and radionuclide decay. Of note, four memory clinic participants were missing [^11^C]PiB-PET data, and these participants were excluded only from the analyses that included Aβ load.

### PET data analyses

Using the structural MPRAGE, we defined six hand-drawn regions, including the frontal cortex (ventral and dorsal), anterior cingulate (subgenual and pregenual), anteroventral striatum, mesial temporal (hippocampus and amygdala), precuneus/posterior cingulate (ventral, middle, and dorsal), parietal cortex, lateral temporal, occipital (calcarine and pole), and cerebellum (49). As described in a prior study (50), PET-MR co-registration was performed using automated image registration algorithms for alignment and interpolation.

We then inspected the dynamic [^11^C]-PiB acquisition frames for interframe motion. If suspected, the automated image registration algorithm, which was optimized for PET-to-PET registration, was applied on a framewise basis. A summed image over the post-injection interval was computed, and a spatial transformation was applied, which was resliced in MPRAGE space. Regional concentrations were then normalized to non-specific uptake in the cerebellum, which yielded a standardized uptake value ratio (SUVR) measure (51). We then partially corrected regional SUVRs using a previously validated method that corrected for the dilution of PET signal due to limited spatial resolution (51–54)—this two-component approach corrects for the dilutional effect of expanded cerebrospinal fluid spaces that accompanies normal aging and disease-related cerebral atrophy using FSL software (University of Oxford, Oxford, UK). We then computed a global PiB retention index reflecting cerebral amyloid load (i.e., Aß load) from a weighted average of the SUVR values from the six regions listed above.

### MRI data acquisition

3T Siemens Trio TIM scanner with a 12-channel head coil was utilized to collect all MRI data for this study. Whole-brain sagittal 3D magnetization prepared rapid-acquisition gradient echo (MPRAGE) sequence parameters included echo time (TE) = 2.98 ms, repetition time (RT) = 2,300 ms, flip angle (FA ) =90^o^, FOV = 256 × 240, 1 × 1 × 1.2 mm resolution, 0.6 mm gap, and a generalized autocalibrating partial parallel acquisition (GRAPPA) acceleration factor of 2. A whole-brain axial EPI (echo-planar imaging) BOLD sequence during the DSST task collected the following parameters: TE = 32 ms, TR = 2,000 ms, FA = 90^o^, FOV = 128 × 128, 2 × 2 × 4 mm resolution with no gap, and GRAPPA factor of 2. Due to low coverage and placement, the MRI scans did not cover the inferior aspect of the cerebellum or the most superior portion of the motor/supplemental motor cortex.

### In-scanner digit-symbol substitution task

Participants completed the computerized version of DSST (28, 29) in the MRI scanner with two conditions: experimental and control. During the experimental condition, a visual cue (1.2 s) that included a simple geometric symbol (e.g., inverted “T”) and a number. After a brief delay (blank screen, 0.2 s), participants were shown four different symbol–number pairs on a single screen. Participants pressed the right button (right-index finger) if the answer key contained the same symbol-number pair as the cue or the left button (left-index finger) if it did not. All participants familiarized themselves with the task before getting into the scanner and were instructed to respond “as fast and accurately as you possibly can.

The control condition was identical to the experimental condition except the answer keys consisted of four of the same symbols with a cue paired by a letter “R” or “L” to press the right or left button, respectively. The task was a block design with eight trials per block. Experimental and control conditions alternated. Each block of the condition (56 s) was repeated five times (a total of 9 min and 20 s).

The main behavioral measure of interest was reaction time during the experimental block (i.e., median in-scanner reaction time for correct trials). This performance index was designed to represent the cognitive processes that align with the subjective experience of cognitive difficulties despite successful task performance. We computed the median reaction time of the trials in the experimental blocks after discarding missing or incorrect trials. Additionally, accuracy and the percentage of missing trials (for each condition separately) were examined to evaluate valid task effort. Two participants with excessive missing trials (more than 90%) in the experimental condition were excluded. Reaction time was correlated with accuracy (*r* = −0.42, *p* < 0.001); however, this cognitively unimpaired cohort had high task accuracy near the ceiling, so reaction time was the more sensitive measure of subtle individual differences in task performance.

### MRI image processing

The structural and functional MRI data in this study were processed using the Statistical Parametric Mapping (SMP12) toolbox in MATLAB 2016b (MathWorks). After co-registration with the MPRAGE image, the FLAIR and T2-weighted sequences were bias-corrected and then segmented into tissue classes for the generation of a deformation field, followed by the normalization of images to Montreal Neurological Institute (MNI) space. An automatic intracranial volume mask was then generated using a 0.1 threshold on the gray matter/white matter/CSF, and the skull was removed after image filling and closing in MATLAB applied to the MPRAGE.

After this, the functional MRI data underwent motion correction, co-registration to the skull-stripped MPRAGE, normalization to MNI space at a 2 mm isotropic resolution, and smoothing with a Gaussian kernel of FWHM 8 mm. Five summary measures of motion were computed using ArtRepair toolbox (http://cibsr.stanford.edu/tools/human-brain-project/artrepair-software.html). Median and interquartile range (in parentheses) values for the following measures were minimal: the maximum range of translational motion 1.67 (1.4), maximum translational motion 1.36 (1.1), average root mean square motion 1.41 (1.1), average scan-to-scan motion across sessions 0.19 (0.1), and percentage of TRs with head jerks (>0.5 mm for combined translation and rotation) 2.14 (6.3). Framewise displacement was computed based on these six motion parameters as a summary score of motion (55) using the “fd” function in the “FIACH” R package (http://search.r-project.org/library/FIACH/html/fd.html).

The effects of the experimental and control conditions were modeled by convolving boxcars of their stimuli with the hemodynamic response function. Additionally, the signal mean, six motion correction parameters, a high-pass filter of 1/128 Kz for signal drift, and an autoregressive filter (for serial correlations due to aliased biorhythms/unmodeled activity) were modeled as well. The contrast between experimental and control was computed for use in group-level analysis.

### Statistical analysis

We utilized statistical non-parametric mapping (SnPM13) toolbox (http://www.nisox.org/Software/SnPM13/) for voxel-wise statistical analysis and multiple comparisons correction (56). We performed voxel-wise permutation testing (5,000 permutations) and used a cluster-forming *p*-value of 0.001 based on Eklund et al. (57).

To identify regions significantly active during the experimental compared to the control condition, we performed a voxel-wise one-sample *t*-test on the experimental-control contrast and corrected the false discovery rate (FDR) rate <0.05. All subsequent voxel-wise analyses were limited to regions that were significantly activated during this task.

To test our main hypothesis, we conducted a voxel-wise regression to evaluate the association between DSST activation (experimental minus control) and SCD symptom severity. We controlled for multiple comparisons by controlling the cluster family-wise error (FWE) at <0.05 because the analysis was limited to only regions significantly active during the task. We also conducted the same voxel-wise regressions to test the associations between DSST activation and task performance (in-scanner reaction time) and between DSST activation and Aβ load.

To test the robustness of activation associated with SCD symptom severity to nuisance variables, we ran a regression analysis in R with extracted mean activation from significant clusters (the outcome variable) with SCD symptom severity (the predictor variable of interest) adjusted for age, sex, race, education, recruitment source (memory clinic vs. community study), global Aβ load, neuroticism, depressive symptoms, task performance (measured by in-scanner reaction time), and framewise displacement motion summary.

## Results

**Figure 1:**
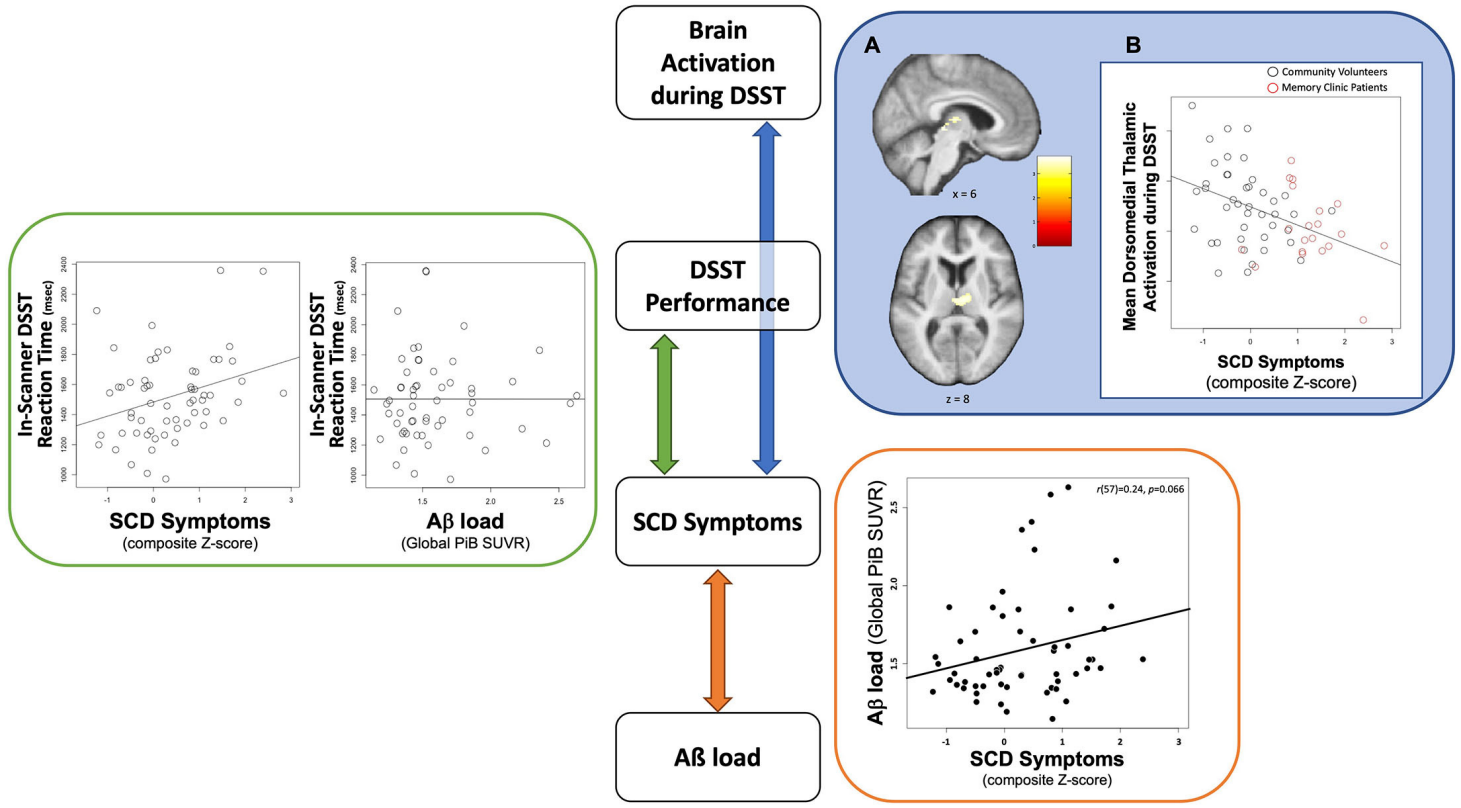
Schematic summary of the cross-sectional findings to highlight relationships among contributing factors to brain activation during DSST among individuals with SCD symptoms. GREEN section: Slower in-scanner DSST reaction time was associated with greater SCD symptoms **(right)** but not Aβ load **(left)**. The association between reaction time and SCD symptoms remained significant when adjusting for demographic factors (age, sex, race, and education). BLUE section: **(A)** Neural correlates of SCD Symptoms in the Dorsomedial Thalamus. Significant cluster (a cluster-FWE *p* < 0.05) in the dorsomedial thalamus overlaid on an average structural brain from all participants. Color-bar indicates value of t-statistic associated with regression term. **(B)** Association between SCD Symptoms and Dorsomedial Thalamic Activation. Plot demonstrating association between lower mean dorsomedial thalamus activations and greater SCD symptoms. Black circles represent community volunteer participants. Red circles represent memory clinic patients. ORANGE section: A positive association between Aß load and SCD symptom severity from the previously published the same sample (30). The scatter plot was recreated with the current sample.

Table 1 summarizes the demographic characteristics of the sample, as well as SCD and neuropsychological scores and in-scanner task performance (detailed sample descriptions are in Supplementary Table S1). The mean age of participants was 73.6 (SD = 7.2), 63% were females, and 90% were Caucasians. The mean years of education of 15.7 (SD = 3.1) indicate a college level of education. In terms of genetic risk, 22% of participants had at least one ε4 APOE allele (with a breakdown of 23% for community volunteers and 21% for memory clinic participants, accounting for three and one instance of missing data, respectively). There was no significant difference in SCD symptom severity observed between participants carrying at least one ε4 APOE allele and those without [*t*_(22.8)_ = 0.13, *p* = 0.90, Welch's *t*-test]. Participants had high (average 94.0%) task accuracy. Slower in-scanner DSST reaction time was associated with greater SCD symptom severity (β = 0.34, *p* = 0.01) but not Aβ load [β = −0.09, *p* = 0.50; *R*^2^ = 0.11, *F*_(2, 55)_ = 3.46, *p* = 0.04; Figure 1: GREEN section]. The association between reaction time and SCD symptom severity remained significant when adjusting for demographic factors (age, sex, race, and education; Table 2).

**Table 1:** Demographic characteristics, neuropsychological test battery and other measures.

**Variables**	**63 Participants (female 63%)**			
	**Mean**	**Median**	**SD**	**Range (min, max)**
Age (years)	73.6	74	7.2	52, 93
Race	57 Caucasian (90%), 4 African American, 1 Asian, 1 not reported			
Education (years)	15.7	16	3.1	12, 24
Aβ (Global PiB SUVR)	1.59	1.47	0.33	1.14, 2.63
MFQ	283.0	285	49.7	139, 398
CFQ	39.2	40	14.0	9, 74
SCCS	5.9	5	4.5	0, 17
SCD symptoms (composite *Z*-score)	0.33	0.24	0.92	−1.23, 2.83
Geriatric Depression Scale	5.3	4	5.5	0, 26
Neuroticism	17.8	17	7.8	2, 42
Mini-mental state examination	29.0	29	1.2	24, 30
**In-scan DSST reaction time (correct trials)**				
Experimental (ms)	1,512	1,489	281.5	973, 2,407
Control (ms)	1,021	965	227	659, 1,768
**In-scan DSST accuracy**				
Experimental (%)	94.0	97.0	11.8	21, 100
Control (%)	98.9	100.0	2.2	90, 100
**In-scan DSST missing trials**				
Experimental (%)	9.7	5.0	14.1	0, 65
Control (%)	5.3	0.0	11.3	0, 50

DSST, digit-symbol substitution task; SUVR, standardized uptake value rate; MFQ, Memory Functioning Questionnaire; CFQ, Cognitive Failures Questionnaire; SCCS, Subjective Cognitive Complaint Scale.

**Table 2:** Regression analysis summary: association between task performance (in-scanner reaction time) and SCD symptoms with covariates of interest.

	**β**	**95% CI**	***t*-value**	***p*-value**
SCD symptoms	0.32	0.00, 0.64	2.13	0.04
Aβ load	−0.08	−0.36, 0.21	−0.49	0.63
Age	0.13	−0.18, 0.43	0.83	0.41
Sex (male)	−0.18	−0.73, 0.36	−1.32	0.19
Race (Asian)	0.01	−2.39, 2.40	0.03	0.98
Race (Caucasian)	0.15	−0.88, 1.18	0.95	0.35
Education	0.08	−0.27, 0.43	0.49	0.63

Standardized β-values are reported.

We found greater activation during the experimental condition (compared to the control condition, Table 3) in the primary visual processing area (calcarine), executive control network (dorsolateral prefrontal cortex, inferior and superior parietal lobes, and precuneus), salience network (insula and dorsal cingulate cortex/supplemental motor area), and subcortical regions (hippocampus and dorsomedial thalamus; Supplementary Figure S2).

**Table 3:** DSST main effect (experimental > control).

**Regions**	**# of voxels**	**Peak intensity (t)**	**MNI coordinates**		
			***x***	***y***	***z***
Calcarine/precuneus/superior parietal lobe (SPL)/inferior temporal (BA37)	8,210	11.4	−30	−84	22
L Hippocampus	22	4.9	−28	−30	−6
R Insula	58	5.3	32	24	0
L Insula	187	5.4	−30	18	8
L Thalamus	200	5.8	−14	−6	10
L Precentral/ IFG	1,837	4.8	−44	2	32
R IFG_Oper	109	5.7	38	4	32
Dorsal anterior cingulate cortex (dACC)/supplemental motor area (SMA)	491	8.6	−6	14	50
R Precentral (BA 6)/IFG	170	5.8	32	0	46

Voxel-wise FDR p <0.05, minimum cluster size = 20.

Greater SCD symptom severity was associated with lower DSST-induced activation in the bilateral dorsomedial thalamus (cluster-wise FWE *p* < 0.05, Figure 1: BLUE section). This association was robust to covariates (age, sex, race, education, recruitment source, global Aβ load, neuroticism, GDS, in-scanner DSST reaction time, and framewise displacement motion; Table 4). To evaluate a possible effect driven by outliers, we repeated this analysis using the non-parametric Spearman's rank correlation. The result confirmed the significant association between SCD symptoms and DSST-induced activation in bilateral dorsomedial thalamus [*r*__*s*_(63)_ = −0.38, *p* = 0.002].

**Table 4:** Regression analysis summary: association between DSST-related activation of the bilateral dorsomedial thalamus and SCD symptom severity, with covariates of interest.

	**β**	**95% CI**	***t*-value**	***p*-value**
SCD symptom severity	−0.53	−1.02, −0.04	−2.58	0.01
Age	0.26	−0.07, 0.60	1.73	0.09
Sex (male)	0.08	−0.48, 0.65	0.62	0.54
Race (Asian)	0.07	−2.48, 2.61	0.40	0.69
Race (Caucasian)	0.13	−0.95, 1.21	0.87	0.39
Education	−0.12	−0.48, 0.24	−0.83	0.41
Recruitment source (memory clinic)	0.31	−0.71, 1.34	1.45	0.16
Aβ load	0.01	−0.27, 0.30	0.08	0.96
Neuroticism	0.19	−0.23, 0.61	0.95	0.34
Geriatric Depression Scale	−0.16	−0.70, 0.37	−0.64	0.53
In-scanner DSST reaction time	0.27	−0.05, 0.59	1.83	0.07
Framewise displacement motion summary	−0.38	−0.66, −0.10	−2.71	0.01

Standardized β-values are reported. In this *post-hoc* analysis, the p-value for the framewise displacement motion summary exhibited the statistical significance (p = 0.01); however, the SCD symptom severity was not associated with framewise displacement motion summary [r_(61)_ = 0.13, p = 0.29].

We found no significant associations between DSST-induced activation and in-scanner DSST reaction time. Aβ load was not associated with either DSST activation or task performance.

## Discussion

We investigated brain activation during an executive function task among older adults with varying levels of SCD symptom severity by using a computerized DSST task that taps working memory and attentional control. Contrary to our hypothesis, we found a negative association between SCD symptom severity and DSST-induced activation in the subcortical region. Greater SCD symptom severity was associated with lower dorsomedial thalamus activation during DSST. The analyses of in-scanner DSST task performance showed no association between DSST-induced activation and reaction time. However, worse task performance (i.e., slower reaction time for correctly answered trials) was associated with greater SCD symptom severity. Task performance was not associated with Aß load. We have previously reported that greater SCD symptom severity was associated with greater Aβ load in the same participants (30). These collective results suggest that neural function and executive task performance are more directly associated with SCD symptom severity than Aβ load.

Self-perceived cognitive decline and concerns likely represent metacognitive awareness (i.e., reflection on and knowledge about one's own cognitive process). Subjective cognitive concerns may arise due to subtle changes in the usage of neural resources and underlying neurobiological changes in the aging brain. The cognitive control network is the typical neurofunctional structure that mediates executive function and associated processes (16). The present study observed the expected task-induced activations in the regions that belong to this network. However, we found the dorsomedial thalamus to be the neural correlate of SCD symptom severity during the executive function task. Recent studies have proposed the contributions of the dorsomedial thalamus to complex cognitive operations by emphasizing its anatomical connection to the frontal cortex (58, 59). The dorsomedial thalamus has reciprocal connections with the prefrontal cortex, including the dorsal anterior cingulate [e.g., studies in both animal (60) and human studies (61)]. This “trans-thalamic” pathway connecting the medial temporal lobe and prefrontal cortex may provide the function of orchestrating the *temporal fidelity* to execute cognitive operations (59, 62). The DSST fMRI paradigm requires coordinating a sequence of temporally discrete information steps: encoding a cue, holding it in working memory with a short delay, identifying the matched symbol while eliminating distractors, and coordinating visuomotor information to choose the correct key. Therefore, lower dorsomedial thalamus activation may indicate that suboptimal trans-thalamic cognitive processes, which are not manifest as an overt impairment (i.e., low accuracy), underlie SCD symptoms.

Working memory is a key component of cognitive operations during DSST performance (27), and our computerized version of DSST is particularly designed to induce working memory activities. To recognize the symbol–number pair that is held as a cue in working memory, there are two possible processing routes: recollection and familiarity (63). Although these two processing routes are mainly investigated in the context of episodic (long-term) memory, the same dual processes are also speculated to be present in working memory and short-term memory (64–66). Recollection refers to the retrieved details of previous experience associated with a given item, whereas familiarity is a mere sense of the previous exposure to a given item and is thought to be mediated in the perirhinal cortex through the dorsomedial thalamus pathway (67–69). The DSST (our version and others) does not require recollection of the elaborate details that were associated with the cue (e.g., when/where the cue was acquired), so familiarity-based memory may be a more likely processing route. Earlier studies have suggested that the process of aging is associated with impaired recollection but not familiarity (70). However, a growing number of studies support the notion that familiarity-based memory is also associated with healthy aging and may be more closely associated with AD-related processes (71, 72). The decline of familiarity-based memory may be a possible means to identify those at risk for developing Alzheimer's disease (71). We suggest that the association between lower thalamic activation and greater SCD symptoms may indicate deteriorating familiarity-based working memory function at an early stage of cognitive decline.

While in-scanner DSST performance showed no direct association with neural activation, worse performance was associated with greater SCD symptom severity (Figure 1: GREEN section). Furthermore, there was no association between task performance and Aβ load. However, we previously reported a positive association between Aß load and SCD symptom severity among these same participants (30) (Figure 1: ORANGE section). With our core finding of the association between brain activation and SCD symptom severity (Figure 1: BLUE section), we suggest that SCD symptoms act as a unifying factor connecting all the variables we tested: Aß load, task performance (reaction time), and brain activation (Figure 1). In the visual summary of results related to this cohort, accumulating Aß load was related to elevated SCD symptom severity (Figure 1: an orange arrow); however, Aß load itself was not related to DSST task performance or brain activation. Instead, we posit that the contribution of Aß load to executive function at the behavioral and neural levels is via SCD symptom severity. It is pertinent to point out that this indirect contribution through SCD symptoms could be specific to our task and cohort, warranting further investigation to better understand the relationship between Aß and SCD.

Collectively, our results underscore the significance of SCD symptoms as an early sign of AD-associated cognitive decline as they not only appeared to reflect Aß load but also deteriorating neural function. In addition to Aß, the relationship of other AD neuropathologies with SCD symptoms needs to be further investigated. In one SCD study, CSF biomarkers of total tau pathology but not Aß were associated with cognitive decline (73). As seen in studies in individuals with MCI, tau pathology may have a direct association with cognitive function in SCD. Although the relationship between Aβ load and SCD is somewhat inconsistent (74, 75), SCD symptoms may be at least partially accounted for by Aβ-associated neural alterations. A more recent study (76) reported that Aβ load was not associated with the general severity of SCD but rather with elevated SCD-related worry and awareness of memory deficit. These suggest that SCD symptoms may encompass broad aspects of metacognitive awareness of not only Aβ-associated cognitive decline but also associated psychological factors, including depressive symptoms, worry, and neuroticism (77).

## Limitations

In this cross-sectional fMRI study, it is unclear how to interpret “low” activation in the thalamus. We lack data on neural function prior to the onset of SCD symptoms, which limits interpretation. The present study employed the approach of investigating SCD symptoms as a continuous variable (i.e., SCD symptom severity) rather than a categorical/diagnostic group in combined clinical and community-dwelling samples. First, the continuous variable approach has been used in SCD research (2, 32, 78), and this approach avoids an arbitrary operationalization of SCD as a diagnostic classification. Second, our approach to investigate combined clinical and community-dwelling samples allowed us to capture neurobiological features that were represented in a wider population (i.e., higher generalizability, not limited to clinical/help-seeking SCD samples). This approach has been previously used in an fMRI study in SCD (79), and it is concordant with recent rising efforts to understand the SCD symptoms among the understudied community-dwelling sample (80–83). Despite various advantages of treating SCD as a continuous variable, there may be qualitative differences between SCD symptoms in memory clinic patients and “questionnaire-discovered” individuals who have SCD symptoms in community samples. We attempted to address this point by analytically controlling for recruitment sources in the present findings. We also have a limited sample size; however, our study has one of the largest samples of those with SCD and both fMRI and amyloid PET (10–12). Considering the known concerns regarding the heterogeneity of individuals with SCD, we need longitudinal studies to interpret inferences about changes accurately. Future studies should also include younger participants (e.g., 50s) to further understand the neural basis of earlier cognitive decline.

## Summary

We found that lower dorsomedial thalamus activation during an executive function task was associated with subjective decline/concerns in older adults. The self-perceived decline in memory and other cognitive function may be clinically informative because these symptoms may reflect an individual's own longitudinal trajectory of cognitive capabilities. Future studies should not be limited to the investigation of the memory domain but consider the subtle decline of executive functions as a neural feature of SCD.

## Data availability statement

The raw data supporting the conclusions of this article will be made available by the authors, without undue reservation.

## Ethics statement

The studies involving humans were approved by the University of Pittsburgh Institutional Review Board. The studies were conducted in accordance with the local legislation and institutional requirements. The participants provided their written informed consent to participate in this study.

## Author contributions

Study conception and design: AM, HK, HA, and BS. Data acquisition: HK, BL, AC, CM, WK, HA, and BS. Analysis and interpretation of data: AM, HK, ML, BL, HA, and BS. Drafting and critical revision: AM, HK, ML, HA, and BS. All authors contributed to the article and approved the submitted version.
