# Supplementary material for: Low thalamic activity during a digit-symbol substitution task is associated with symptoms of subjective cognitive decline
Source: Front Psychiatry. 2023 Sep 6;14:1242822. doi: 10.3389/fpsyt.2023.1242822 (PMC10511647; doi:10.3389/fpsyt.2023.1242822)
Supplement: Supplementary file 1 [file Data_Sheet_1.docx]

Supplemental Materials

**Supplemental Table S1**

|  | |  | | Community Volunteers | | Memory Clinic | | |  |  |
| --- | --- | --- | --- | --- | --- | --- | --- | --- | --- | --- |
|  |  |  |  | n=43 (65% female) | | n=20 (60% female) | | |  |  |
| Variables | | | | mean | sd | | mean | sd | *t*-test | |
| Age (years) | | | | 76.3 | 6.4 | | 67.7 | 5.1 | *t*(46) = 5.81 | *p* < 0.001 |
| Education (years) | | | | 14.6 | 2.4 | | 18.2 | 3.1 | *t*(31) = -4.60 | *p* < 0.001 |
| Aβ (Global PiB SUVR) | | | | 1.57 | 0.3 | | 1.62 | 0.36 | *t*(25) = -0.46 | *p* =0.65 |
| SCD Symptoms | | | | -0.1 | 0.7 | | 1.3 | 0.7 | *t*(35) = -7.35 | *p* < 0.001 |
|  | | MFQ | | 302.1 | 42.8 | | 243.7 | 39.1 | *t*(41) = 5.31 | *p* < 0.001 |
|  | | CFQ | | 32.8 | 10.6 | | 52.5 | 10.3 | *t*(39) = -6.95 | *p* < 0.001 |
|  | | SCCS | | 3.9 | 3.3 | | 10.1 | 3.7 | *t*(34) = -6.24 | *p* < 0.001 |
| Geriatric Depression Scale | | | | 2.8 | 2.9 | | 10.9 | 5.8 | *t*(22) = -5.77 | *p* < 0.001 |
| Neuroticism | | | | 15.4 | 5.6 | | 23.9 | 9.1 | *t*(24) = -3.76 | *p* = 0.001 |
| Global Cognitive Function | | | |  |  | |  |  |  |  |
|  | Mini-Mental State Examination | | | 29.0 | 1.2 | | 29.0 | 1.2 | *t*(36) = -0.07 | *p* = 0.95 |
| Memory | | | |  |  | |  |  |  |  |
|  | Modified Rey-Osterrieth Complex Figure | | |  |  | |  |  |  |  |
|  | |  | Immediate recall | 17.2 | 3.0 | | 19.3 | 3.0 | *t*(34) = -2.37 | *p* = 0.03 |
|  | |  | Delayed recall | 17.0 | 3.0 | | 19.0 | 3.2 | *t*(23) = -2.12 | *p* = 0.05 |
| Language | | | |  |  | |  |  |  |  |
|  | Modified Boston Naming Test | | | 28.7 | 2.0 | | 28.8 | 1.5 | *t*(30) = -0.08 | *p* = 0.93 |
|  | Letter/Category Fluency | | | 47.3 | 14.0 | | 47.1 | 16.4 | *t*(24) = 0.05 | *p* = 0.96 |
| Visuospatial Abilities | | | |  |  | |  |  |  |  |
|  | Modified Block Design | | | 14.0 | 4.1 | | 15.7 | 3.8 | *t*(29) = -1.45 | *p* = 0.16 |
| Executive Function | | | |  |  | |  |  |  |  |
|  | Trail Making Test (sec) | | |  |  | |  |  |  |  |
|  | |  | A | 29.8 | 9.9 | | 27.4 | 7.5 | *t*(45) = 1.07 | *p* = 0.29 |
|  | |  | B | 84.5 | 38.0 | | 65.6 | 18.7 | *t*(59) = 2.63 | *p* = 0.01 |
|  | Digit-Symbol (Out-Scanner) | | | 52.1 | 12.6 | | 50.7 | 9.3 | *t*(37) = 0.47 | *p* = 0.64 |
| In-Scan DSST Reaction Time | | | |  |  | |  |  |  |  |
|  | | Experimental (msec) | | 1454 | 259 | | 1649 | 281 | *t*(35) = -2.61 | *p* = 0.01 |
|  | | Control (msec) | | 997 | 211 | | 1077 | 250 | *t(*33) = -1.22 | *p* = 0.23 |
| In-Scan DSST Accuracy | | | |  |  | |  |  |  |  |
|  | | Experimental (%) | | 95.0 | 6.9 | | 91.9 | 18.2 | *t*(22) = 0.74 | *p* = 0.47 |
|  | | Control (%) | | 98.6 | 2.6 | | 99.3 | 1.3 | *t*(59) = -1.36 | *p* = 0.18 |
| In-Scan DSST Missing Trials | | | |  |  | |  |  |  |  |
|  | | Experimental (%) | | 11.9 | 13.6 | | 5.1 | 14.3 | *t*(36) = 1.79 | *p* = 0.08 |
|  | | Control (%) | | 6.6 | 13.1 | | 2.8 | 5.2 | *t*(57) = 1.63 | *p* = 0.11 |

**Supplemental Figure S1**

Abbreviation: SCD: Subjective Cognitive Decline, MFQ: Memory Functioning Questionnaire, CFQ: the Cognitive Failures Questionnaire, SCCS: Subjective Cognitive Complaint Scale

**Supplemental Figure S2**

DSST Main Effect (Experimental > Control) (voxel-wise FDR *p*< 0.05)
